# The Frequency of Chromosomal Euploidy Among 3PN Embryos

**Published:** 2019

**Authors:** Kresna Mutia, Budi Wiweko, Pritta Ameilia Iffanolida, Ririn Rahmala Febri, Naylah Muna, Oki Riayati, Shanty Olivia Jasirwan, Tita Yuningsih, Eliza Mansyur, Andon Hestiantoro

**Affiliations:** 1- Human Reproductive, Infertility and Family Planning Research Center, Indonesian Medical Education and Research Institute (IMERI), Faculty of Medicine, Universitas Indonesia, Jakarta, Indonesia; 2- Division of Reproductive Endocrinology and Infertility, Department of Obstetrics and Gynecology, Faculty of Medicine, Universitas Indonesia, Jakarta, Indonesia; 3- Yasmin IVF Clinic, Dr. Cipto Mangunkusumo General Hospital, Jakarta, Indonesia

**Keywords:** Aneuploidy, Embryo, IVF, Mosaisicm, Pronucleus

## Abstract

**Background:**

The evaluation of embryo morphology is one of the most important parameters used to evaluate developmental timing, also providing an indication of chromosomal failure or degeneration. The first step in the evaluation of a fertilization event is determining the number and shape of the pronuclei (PN). Normally fertilized eggs possess two even PN. However, some embryos which develop from abnormally fertilized zygotes may be tri-pronuclear zygotes (3PN).

**Methods:**

Thirty embryos were collected from 12 women who underwent *in vitro* fertilization (IVF) at Dr. Cipto Mangunkusumo General Hospital in Jakarta, Indonesia. Embryos were cultured until the blastocyst stage on days 5–6. The blastomere biopsy was performed by piercing the zona pellucida with a laser under a microscope. Chromosomal numerical abnormalities were analyzed using Next Generation Sequencing (NGS).

**Results:**

Among the 30 embryos with 3PN zygotes, 33.3% had a normal chromosomal array, with 22 pairs of autosomes and 2 pairs of sex chromosomes. While the rest of sample population detected as abnormal chromosome (66.7%), with the highest percentage of abnormality was triploidy 43.3%, followed by mosaicism 13.4% and aneuploidy 10%.

**Conclusion:**

This was a preliminary study revealed not all morphologically 3PN embryos are genetically abnormal.

## Introduction

Embryo morphology is an evaluation of embryo quality. Evaluation of embryo morphology is one of the most important parameters to evaluate developmental timing and provide an indication of chromosomal failure or degeneration. The first step in confirming a successful fertilization is determining the number and shape of the pronuclei (PN). The normally fertilized eggs are indicated by 2 even PN (1–3). However, some embryos may develop from abnormally fertilized zygotes like tripronuclear zygotes (3PN). The incidence of 3PN zygotes is 5.0%–8.1% during *in vitro* fertilization (IVF) and 2.5%–6.2% in intracytoplasmic sperm injection (ICSI). The prevalence of 3PN in all pregnancies has been estimated to be approximately 1–3%, whereas it accounts for 15–18% of cytogenetically abnormal cases among spontaneous abortions. Therefore, these zygotes or embryos can’t be used for embryo transfer or cryopreservation, which lowers the number of usable embryos (4, 5).

Abnormal fertilization leading to 3PN formation is a common phenomenon in ICSI and IVF. In early stages of the 3PN zygote development, cellular cleavage is likely to be normal, with arrested development or aneuploidy occurring at a later development stage (3). 3PN formation may be caused by polyspermic fertilization or by oocytes derived from meiotic failure. However, polyspermic fertilization should not occur in ICSI, since only one sperm is injected into each oocyte. Therefore, during IVF, 3PN incidences are generally due to a retention of the second polar body after ICSI, in addition to other factors that have not yet been studied in detail (6, 7).

Previous studies have suggested that embryos with 3PN, which develop from ICSI zygotes, are endowed with mechanisms to correct triploidy to diploidy. 62.5% of ICSI 3PN embryos were found to progress back to the blastocyst stage, whereas 54.5% were heteroparental diploid blastocysts (5). Nowadays, embryo abnormalities can be detected prior to embryo transfer. To obtain the best quality embryos, pre-selection must be done to choose the embryos with the highest implantation potential. The current methods of embryo selection are based on morphology, metabolomics, morphokinetics, and preimplantation genetic diagnostics and preimplantation genetic screening (PGD/PGS) (8,9).

Embryo selection based on morphology alone is not able to ascertain the chromosomal status of the embryo. In a previous study, 50% of embryos with a normal morphology had abnormal chromosomes, and vice versa, including 3PN embryos. Therefore, in the present study, an attempt was made to evaluate the frequency of 3PN morphology embryos that also had a normal chromosomal status.

## Methods

### Sample collection:

Thirty 3PN embryos from patients undergone ICSI-IVF procedure were collected between January–July 2018. All the patients were asked for permission to include their 3PN zygotes in the study. Oral and written informed consent was obtained from the patients. This work was approved by the ethics committee of Komite Etik Penelitian Kesehatan, Universitas Indonesia–Dr. Cipto Mangunkusumo General Hospital, Jakarta, Indonesia.

### Pronuclear examination and embryo biopsy:

Assessment and recording of the pronuclei were performed using an inverted microscope 18–20 hours post ICSI. The morphology of each embryo was recorded and assessed daily based on cell count, percentage of fragmentation, and blastomere size. Embryos that reached the blastocyst stage were assessed using Gardner’s score based on the degree of expansion, intracellular mass (ICM) and trophectoderm (TE). Embryos that reached the blastocyst stage on day 5 or 6 were included in the next phase of analysis.

Embryo biopsy was performed on day 5 or 6 for chromosomal analysis using Next Generation Sequensing (NGS) method. Embryos developed into blastocysts on day 5 or 6 were biopsied on 3–6 TE cells using a laser (Octax, Germany).

### Chromosomal analysis using NGS:

Chromosomal status of each blastomere was determined using VeriSeq library preparation based on the manufacture’s guideline (Illumina, Inc., California, USA). Negative controls from embryology and genetics laboratory were also prepared to make sure there was no contamination in the preparation process. Both blastomeres and negative controls were extracted using 24 Sureplex DNA Amplification Kit from Illumina. All of the samples were processed for library preparation, which consisted of quantification of unpurified Sureplex product, tagmentation, PCR amplification, PCR clean up, library normalization, and library pooling (Illumina Inc). The samples were then sequenced in Miseq NGS (Illumina). This method could screen all the 24 chromosomes including sex chromosomes. The resulting bioinformatics data were analyzed on BlueFuse Software (Illumina Inc.)

### Statistical analysis:

Statistical analysis was performed using SPSS software, version 22.0. Continuous data are presented as the mean (SD) and 95% confidence interval (CI), or medians (min–max). Categorical variables are presented as n (%) and 95% CIs. Data were analyzed by chi-square or Fisher’s exact tests using Odds Ratio (OR). A p<0.05 was considered statistically significant.

## Results

Thirty 3PN embryos were biopsied and chromosomal analysis was performed using NGS. All of the embryos were obtained from 16 patients at Yasmin IVF Clinics-Dr. Cipto Mangunkusumo General Hospital, Jakarta, Indonesia. The general characteristics of the patients are listed in table 1. The mean age of the female patients was 35.56± 2.73 with a duration of infertility of 4.19±2.59 years. The most common causes of infertility were male factors (n=6) consisting of oligozoospermia (n=3), oligoasthenozoospermia (n=2) and teratozoospermia (n=1) cases, followed by polycystic ovary syndrome (PCOS) (n=5), unexplained infertility (n=3) and endometriosis (n=2) cases.

**Table 1.** Characteristics of ICSI-IVF patients with 3PN embryos

**Characteristics**	**Value n=16**
**Female age (years)**	32.56±2.73
**Male age (years)**	36.15±5.80
**Infertility duration (years)**	4.19±2.59
**Infertility causes, n (%)**	
**Male factors**	6 (37.5)
**PCOS**	5 (31.3)
**Unexplained**	3 (18.8)
**Endometriosis**	2 (12.5)
**Oocytes retrieved**	18.19±7.58
**Mature oocytes**	14.69±6.25
**AMH level (*ng/dl*)**	6.53±4.69

From our study, it was found that the frequency of 3PN in each patient was 4.55–46.15%. Among 30 3PN embryos, there were 10 embryos with no aneuploidy detected (33.3%) and 20 embryos were aneuploidy detected (66.7%), most of which were triploidy (43.3%). The distribution of the chromosomal status in 3PN embryos is shown in table 2.

**Table 2.** Distribution of 3PN embryo

**Chromosomal status**	**n**	**%**
**Normal (euploid/diploid)**	10	33.3
**Abnormal**	20	66.7
Aneuploidy	3	10.0
Triploidy	13	43.3
Mosaicism	4	13.3
**Total**	30	100

## Discussion

In the IVF laboratory, pronuclear status, subsequent development rate and embryo morphology are common parameters in the selection of good quality embryos. Previous studies have reviewed the morphological attributes of abnormal pronuclei (10). In the present study, a strong relationship between abnormal pronuclear development and aneuploidy was found, despite the fact that many of the abnormal pronuclear embryos displayed developmental competence. The importance of these early morphological attributes should not be overlooked, and may lead to a more accurate method of embryo selection. Recent studies using PGS to monitor chromosome number suggest that another parameter, ploidy, must also be taken into consideration when choosing embryos with the highest implantation potential.

In this study, the chromosomal array form of the 3PN embryos was analyzed. From our investigation, 33.3% of the 3PN embryos from ICSI-IVF procedures had a normal chromosome. All of the 3PN embryos could be achieved at blastocyst stage on day 5 or 6. 1–4% of human zygotes were found to be 3PN, but there is less information about the chromosomal composition derived from these zygotes because 3PN embryos are generally discarded in routine IVF as they are thought to be genetically abnormal. But some of these 3PN embryos do not always develop into triploid embryos. The study from Kola et al. showed that most of 3PN human embryos do not develop into triploid embryo (11). In another study from Chen et al., they evaluated the chromosomal composition of 3PN embryos and concluded that various amounts of them were diploid (12).

In 3PN zygotes, the prevalence of the normal chromosome could occur at a later stage. However, 3PN zygotes are routinely discarded during the IVF procedure as they are believed to be genetically abnormal. There are various reasons for 3PN fertilization which remain to be fully described. A 3PN zygote is produced when two sperm enter the same oocyte during IVF, or if the second polar body is not expulsed during ICSI. Despite increased evidence that abnormal spermatozoa from infertile men can be a risk factor in ICSI treatment, the extent to which these abnormalities might contribute to chromosome irregularities at the time of conception is still unknown (2, 13). 3PN could also be produced by oocyte-derived meiotic failure. Furthermore, some studies have suggested that 3PN fertilization after IVF is a result of advanced maternal age or severe sperm abnormalities, while other studies have suggested that 3PN may occur due to ovarian stimulation which is accompanied by high peak E2 levels, large oocyte yields, high gonadotropin doses, and lengthy stimulation procedures (14, 15). Chromosomal abnormalities are responsible for a high proportion of embryonic loss, as they cause slow-cleavage rates in embryos. From previous study, it is known that aneuploid embryos are more likely to be developmentally blocked on day 3 and days 5–6 than those diagnosed as euploid (16).

**Figure 1.**
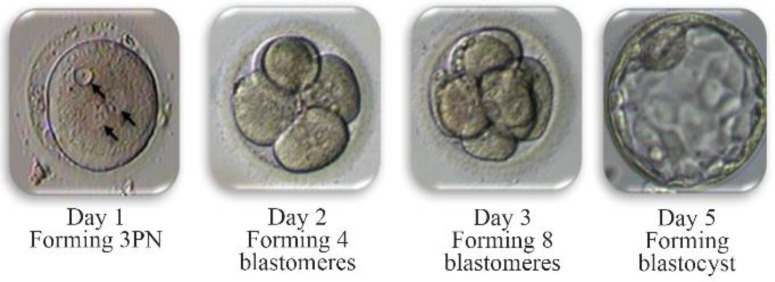
The development of 3PN embryos

In the present study, 33.3% of 3PN embryos had no chromosomal abnormalities, and had 46, XX. All of the embryos were cultured until the blastocyst stage (Figure 1). This implies that a natural selection mechanism may occur, where chromosomally abnormal embryos are eliminated before blastocyst formation or later, prior to or shortly after implantation. This condition would be associated with the presence of an abnormal chromosomal complement in the vast majority of the cells constituting the embryo. If the number of aneuploid cells does not exceed a certain limit, they are diverted to the trophectoderm, whereas normal cells are allocated to the inner cell mass (ICM) (17, 18).

**Figure 2.**
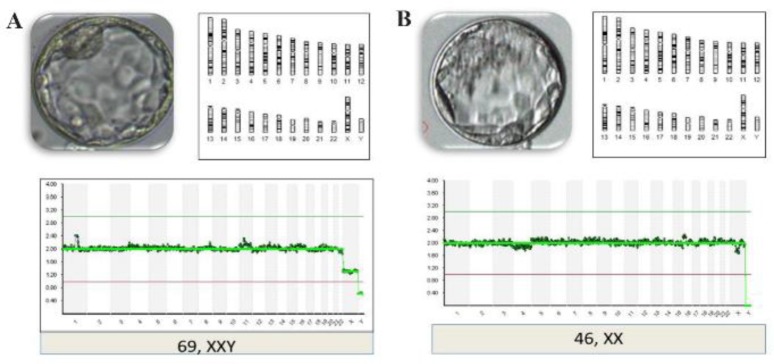
NGS profile of 3PN embryos

Among the 66.7% of chromosomally abnormal 3PN embryos, all of them were 69, XXY, and 13.3% of them showed mosaicism. From previous studies, it is known that aneuploidy and mosaicism in 3PN embryos can still constitute “normal” developmental events, with an etiology as mentioned above, and not necessarily a consequence of the 3PN status. Although it has been demonstrated that 3PN zygotes generally have 3 haploid sets of chromosomes, several studies of 3PN embryos have revealed triploid, diploid, or severely abnormal arrays (Figure 2). As shown in table 2, most of the 3PN embryos were found to be perfect triploids. The differences in the percentage of diploid embryos could be caused by the presence of a misleading vacuole. None of the previous studies examined XYY embryos, with equal numbers of XXX and XXY, again indicating that the mechanism was digyny. This indicates that chromosomes in 3PN zygotes were organized in a single bipolar spindle at syngamy, and suggests that only one centrosome is active (3).

In IVF procedures worldwide, abnormal embryos are routinely discarded due to their increased risk for abnormalities. Different studies have demonstrated the reproductive potential of abnormal embryos; for example, healthy babies born from 1PN fertilized oocytes have been reported (19–21). Feng and Herbert (2006) concluded that 3PN embryos have 61.8% of triploid chromosome complement, 25.2% of mosaic arrangements, and only 12.65 of them had a diploid chromosome (22). There is evidence of naturally occurring triploid pregnancies but most of them result in miscarriage and neonatal deaths (23). The abnormalities in 3PN embryos are rising, which include intrauterine growth retardation, major central nervous system defect and abdominal wall defect. Contrary to the study of Ender et al. in 2016, a healthy baby from 3PN embryo was reported (24).

The genetic data from single blastomere may not be representative of the one for the whole embryo. It could be caused by high ratio of mosaicism in preimplantation day 3 embryos. It has also been the limitation of this study, because the timing of blastomere biopsy was not accurate. For further studies, biopsy day should be set on day 5 on a larger number of 3PN embryos to minimize the confounding effect of mosaicism.

## Conclusion

In conclusion, our study reveals that blastocyst morphology has a strong association with the potential for chromosomal abnormalities in 3PN embryos, and that not all 3PN embryos have genetic abnormalities. Additional studies using a larger number of embryos will be required to confirm these results.
